# DNA methylation differs extensively between strains of the same geographical origin and changes with age in *Daphnia magna*

**DOI:** 10.1186/s13072-020-00379-z

**Published:** 2021-01-06

**Authors:** Jack Hearn, Fiona Plenderleith, Tom J. Little

**Affiliations:** 1 Department of Vector Biology, Liverpool School of Tropical Medicine, Liverpool, UK; 2 The James Hutton Institute, Craigiebuckler, Aberdeen, UK; 3 School of Biological Sciences, University of Aberdeen, Aberdeen, UK; 4 Institute of Evolutionary Biology, School of Biological Sciences, University of Edinburgh, Edinburgh, UK

**Keywords:** DNA methylation, Life-history traits, Variation, *Daphnia*, Ageing, Epigenetic

## Abstract

**Background:**

Patterns of methylation influence lifespan, but methylation and lifespan may also depend on diet, or differ between genotypes. Prior to this study, interactions between diet and genotype have not been explored together to determine their influence on methylation. The invertebrate *Daphnia magna* is an excellent choice for testing the epigenetic response to the environment: parthenogenetic offspring are identical to their siblings (making for powerful genetic comparisons), they are relatively short lived and have well-characterised inter-strain life-history trait differences. We performed a survival analysis in response to caloric restriction and then undertook a 47-replicate experiment testing the DNA methylation response to ageing and caloric restriction of two strains of *D. magna.*

**Results:**

Methylated cytosines (CpGs) were most prevalent in exons two to five of gene bodies. One strain exhibited a significantly increased lifespan in response to caloric restriction, but there was no effect of food-level CpG methylation status. Inter-strain differences dominated the methylation experiment with over 15,000 differently methylated CpGs. One gene, Me31b, was hypermethylated extensively in one strain and is a key regulator of embryonic expression. Sixty-one CpGs were differentially methylated between young and old individuals, including multiple CpGs within the histone H3 gene, which were hypermethylated in old individuals. Across all age-related CpGs, we identified a set that are highly correlated with chronological age.

**Conclusions:**

Methylated cytosines are concentrated in early exons of gene sequences indicative of a directed, non-random, process despite the low overall DNA methylation percentage in this species. We identify no effect of caloric restriction on DNA methylation, contrary to our previous results, and established impacts of caloric restriction on phenotype and gene expression. We propose our approach here is more robust in invertebrates given genome-wide CpG distributions. For both strain and ageing, a single gene emerges as differentially methylated that for each factor could have widespread phenotypic effects. Our data showed the potential for an epigenetic clock at a subset of age positions, which is exciting but requires confirmation.

## Background

Many progressive diseases of ageing in humans are associated with epigenetic modifications, the structural modifications on DNA that influence gene regulation. The relationship between epigenetic control and lifespan in humans (and many model systems) is complicated by variation in ageing rates between diet and genotype. The clonally reproducing invertebrate *Daphnia magna* is a tractable model for disentangling these effects. *Daphnia* have lifespans that can be studied within a reasonable time frame (~ 50 to 100 days in the laboratory), different strains are known to show natural variation in longevity, and in response to caloric restriction [1]. We can separate genetic from environmental effects in this species through genetically identical sister-clone replicates. *Daphnia magna* therefore enables experiments that assess the response of DNA methylation to ageing and known lifespan-extending treatments, such as caloric restriction (CR) [2, 3]. Prior to this study, these interactions have not been explored together to determine their influence on DNA methylation.

### Arthropod DNA methylation and inter-genotype diversity

DNA methylation is enriched in exon sequences of various arthropods, including members of holometabolous insect orders Lepidoptera and Hymenoptera [4–7] and species of the Crustacea genus *Daphnia* [8, 9]. Exon-enriched DNA methylation has been shown to occur in genes expressed across multiple tissues [6, 7, 10, 11]. This is not the only pattern of DNA methylation observed in arthropods, however. Hemimetabolous insects, such as cockroaches and termites, and the Crustacean *Crassostrea gigas* have higher genome-wide levels of DNA methylation that do not show exon bias to the extent of holometabolous insects and *Daphnia* [7, 12, 13]. The effect of genetic diversity on patterns of DNA variation has not been assessed in previous arthropod methylation studies. Knowledge of this has come from mammalian studies: in mice, for example, thousands of methylated cytosines (CpGs) have been found to differ between strains [14, 15], which was associated with proximity to DNA polymorphisms [14, 16]. Inter-individual polymorphism in DNA methylation has also been observed in humans [17, 18].

### DNA methylation and ageing

Mammals have high levels of CpG methylation genome wide (~ 70%), and degradation of methylation mechanisms are associated with cancers, diabetes and other conditions [19]. Furthermore, it is well established that CpG methylation status can be used to accurately predict chronological age in mammals [20, 21]. This was first discovered in humans, but markers have been developed for a variety of mammals and a bird species [22–26]. Such ‘epigenetic clocks’ can be used to predict acceleration in the ageing process due to smoking or other factors with negative health effects. Dissecting the interactions between ageing and methylation regulation in Arthropods has lagged by comparison to mammals, although pharmacological inhibition (by RG108) of DNA methyltransferase activity was shown to increase lifespan in honeybees [27]. DNA methylation has been correlated to phenotypes in several insect orders: it shows sex-specific patterns in the Hemipteran aphids and mealybugs [28, 29], nurse and forage caste-specificity in honeybees [30], and is essential to larval development in the almost negligibly methylated mosquito species *Anopheles albimanus* [31]. Despite these correlations, causative links between phenotype and DNA methylation have yet to be established in arthropods [7].

### Caloric restriction and DNA methylation

Caloric restriction (CR) is the reduction of dietary intake without malnutrition or loss of micronutrients. It can increase lifespan in many animals, including *D. magna* [32–38]. CR acts through several molecular mechanisms which are possibly master regulated by the mechanistic target of rapamycin (mTOR) pathway [39]. CR in mice causes genome-wide remodelling DNA methylation that delays age-related changes [40]. However, in honeybees lifespan increases were observed on inhibition of DNA methyltransferase, but this was shown to occur in a CR-independent manner [27].

### An epigenomic model invertebrate—*Daphnia magna*

Under favourable ecological conditions [41], *Daphnia* species reproduce clonally. Over time this results in high within-population genetic variation, as multiple non-recombining lineages are maintained [42–45]. Life-history experiments have exploited this variation to explore how distinct genotypes (or strains) of a variety of *Daphnia* species respond to environmental stressors [1, 46–52]. *D. magna* has more recently emerged as an epigenetic model organism. A variety of life-history and ecotoxicological stimuli have been shown to modify methylated cytosines (CpGs), histones and small RNA expression [1, 3, 9, 53–60]. In this role, *Daphnia* species have an advantage over the model invertebrate *Drosophila melanogaster*. *D. melanogaster* has negligible levels of adult DNA methylation and lacks the essential DNA methyltransferase genes (DNMT1 and DNMT3A/B), although this is subject to continuing debate (discussed in [61]). *D. magna* encodes a full complement of DNMTs and gene-body DNA methylation has been shown to correlate with increased gene expression in male versus female *D. pulex* [9]. This is despite low levels of overall cytosine (CpG) methylation of 0.25–0.8% estimated in *D. magna* [8, 53–59], which is sparsely distributed across *Daphnia* genomes [8, 9, 53, 55, 56, 58, 59]. Hence, DNA methylation is likely a directed and non-random process in *Daphnia* that has a role in the regulation of gene expression.

### A multi-factor experimental design

**Fig. 1:**
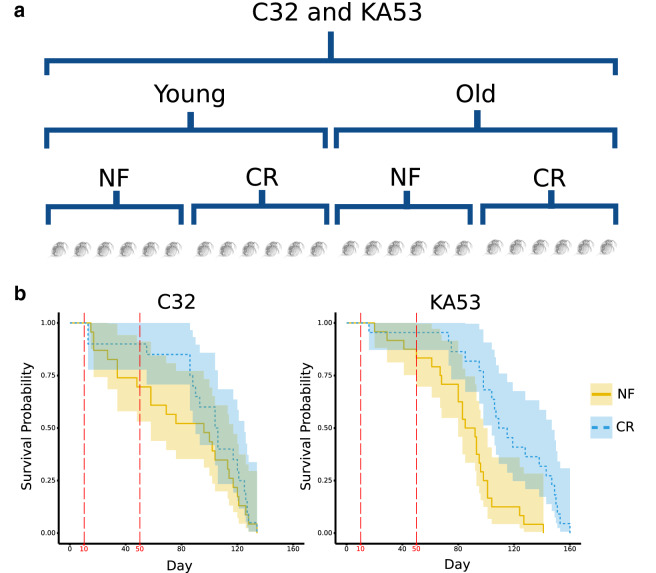
Ageing and response to caloric restriction in strains C32 and KA53. **a** Experimental design for each strain, half of replicates were sampled at 10 days and half at 50 days and half of replicates were submitted to normal food (NF) and half to caloric restriction (CR). This resulted in eight combinations of factors (two strains x two ages x two food levels), each of which consisted of six replicates. **b** Survival curves for C32 and KA53 in response to caloric restriction and normal food levels. NF = normal food; CR = caloric restriction X-axis = time in days; orange line = survival probability for normal food; orange shading = 95% confidence interval of the normal food survival probability; blue dashed line = 95% confidence interval of the caloric restriction survival probability; CR = caloric restriction; red dashed vertical lines indicate sampling points for young replicates at 10 days, and old replicates at 50 days

We have previously shown that CR causes differential methylation, gene and miRNA expression in *D. magna* [1, 3, 59]. However, we found no correlation between differentially methylated regions and gene expression resulting from CR [1], which necessitated confirmation of any link between CR and CpG methylation in this study. This lack of correlation also informed our choice to take a different approach to identifying strain, age and CR changes in CpG methylation here. Firstly, smoothing-based approaches to identify changes in methylation status (as we applied before [59]) combine methylated cytosines in close proximity, were developed with the high levels of DNA methylation in vertebrates as a basis. Secondly, our experimental design is complex incorporating two strains, abundant versus calorically restricted food levels and young versus old individuals (detailed in Fig. 1a). We therefore applied a generalised-linear modelling approach to identify individually differentially methylated CpGs [62]. The advantage of this approach over alternatives (discussed in [54]) is that no assumptions were made about the underlying distribution of DNA methylation, and we were able to assess all experimental factors in one model.

We first conducted a survival analysis of CR on our two strains, which showed that one strain has increased longevity in response to CR but the other does not. Then we tested if genome-wide methylation status varies by Age, CR and Strain. Strain is by far the strongest factor in the experiment accounting for the majority of significant differentially methylated CpGs. Age was significant at much fewer CpGs, but these positions predict epigenetic ages that are highly correlated with chronological age, hinting at the potential for defining an epigenetic clock in *Daphnia*. In contrast to our previous research, CR had no consistent effect on methylation.

## Results

### Strain lifespan and response to CR

The two strains, known to us as strain C32 and KA53, respond differently to caloric restriction (survival curves: Fig. 1b). Median survival time in strain KA53 changed from 89 days (95% confidence interval: 80–98 days) to 112 (95% CI 104–147) with a likelihood ratio test *p*-value of 2 × 10^–4^ in response to CR. For C32, median survival increased from 96 days (95% CI 58–117) to 105 days (95% CI 90–125), but this difference was not significant (*p*-value 0.4, likelihood ratio test).

### WGBS metrics

MultiQC collated mapping, conversion rates and other metrics per replicate are given in Additional files 1 and 2 for the two filtering methods: (1) to control for incomplete bisulphite conversion, reads with three or more consecutive unconverted CH cytosines were removed (‘3x*u*CH’) as recommended by Ref. [63], and (2) all reads containing CH methylated cytosines were removed ( ‘No-CH’) in line with our previous DNA methylation study *Daphnia* [59]. Mapping rates were highly correlated between the reference genome [64] and *D. magna* version 2.4 genome assembly [65], with a Spearman’s rho of 0.997 (*p*-value 2.2 × 10^–16^). The low mapping rates of some replicates (lowest 29.8% reads aligned; highest 66%) do not result from a different alignment reference from our previous study [59]. Average percentage CpG methylation was 1.26% and 1.33% for the ‘No-CH’ and ‘3x*u*CH’ datasets, respectively, (column: ‘% mCpG’, Additional files 1 and 2), while cytosine coverages varied from 8 to 28 reads for both filtered datasets (column: ‘C Coverage’ Additional files 1 and 2).

### Replicates separated clearly by Strain

During Initial data exploration, we discovered that one sample of strain C32 clustered with KA53 (Sample 5, Additional file 3: Figure S1), which we believe to have been a contamination or mislabelling error. This replicate, a strain C32—age Old—food level Normal Food, was removed from the analysis, and all results described were based on analysis of the remaining 47 replicates.

**Fig. 2:**
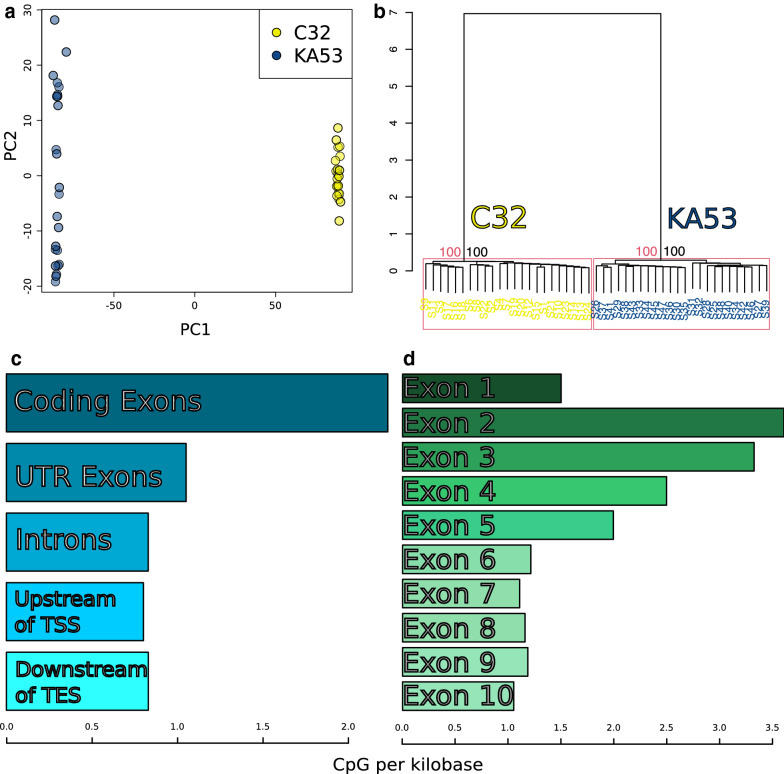
Methylation status correlates with Strain. **a** PCA plot of replicates for principal components one and two for the top 10,000 most variably methylated CpGs by beta value; C32 = yellow, KA53 = blue. **b** Hierarchical clustering of replicates with colouring as for **a**), replicates split by strain with 100% approximately unbiased *p*-value (red) and bootstrap probabilities (black). **c** CpGs with greater than 5% methylation combined across replicates are enriched in the coding regions of exons versus the rest of gene bodies. **d** CpGs with greater than 5% methylation combined across replicates are concentrated in exons two to five in line with previous studies investigating DNA methylation in *D. magna* [8]

PCA and hierarchical clustering of the 10,000 most variable methylated CpG sites both show separation of replicates by Strain (Fig. 2a, b). The first PCA component explained 75.4% of the variance and the second 1.2%, and the hierarchical clustering separated replicates by strain with 100% support from approximately unbiased p-value and bootstrap probabilities. CpGs with greater than 5% methylation were concentrated in coding sequences, with 2.2 CpGs per kilobase versus untranslated regions (1.0 CpGs per kb), introns (0.8 per kb) and transcription start (0.8 per kb) and end sites (0.8 per kb) (Fig. 2c, Tabular results: Additional file 4: Table S1), and there is no difference in these distributions between strains (Additional file 5: Figure S2). When broken down by exon, CpGs occurred more frequently in exons two to five, with the highest frequency in exon two at 3.6 CpGs per kb (Fig. 2 part D, tabular results: Additional file 6: Table S2), and the distribution across exons was not different between strains (Additional file 5: Figure S2).

### Strain explains most significant CpGs

A total of 4,141,957 and 4,095,469 CpGs were input to DSS for the ‘3x*u*CH’ and ‘No-CH’ datasets, respectively. For the ‘3x*u*CH’ dataset, 15,139 significant CpGs were associated with Strain, and 61 CpGs were significant for Age. There were fewer significant sites for the ‘No-CH’ dataset for both factors (Table 1, Additional file 7: Table S3 for significant CpGs per filtering level and factor). No sites were significant for food level, nor were any interaction terms in the ‘3x*u*CH’, ‘No-CH’ and unfiltered datasets. Strikingly, 87% (13,229/15,139 CpGs) of differentially methylated CpGs for Strain in this ‘3x*u*CH’ dataset occur in gene bodies (exons + introns) versus inter-genic sequence. To place this number into context, 49% of the genome assembly (60,352,426 bp out of 122,952,669) is covered by gene body sequence. This was also true for the ‘No-CH’ dataset at 88% (12,282/14,011 CpGs) (list of significant CpGs: Additional file 8: Table S4). Compared to the unfiltered dataset, the results for Strain were very similar to the ‘3x*u*CH’ dataset at 15,139 versus 15,150, but not for Age: 6,978 CpGs were significant in the unfiltered versus the 61 for the ‘3x*u*CH’ dataset (Additional file 7: Table S3).

**Table 1:** Numbers of CpG sites hypermethylated in each strain relative to the other for Strain and Age factors in the ‘3x*u*CH’ and No-CH’ filtered datasets

Strain		
Dataset	Hypermethylated in C32	Hypermethylated in KA53
3x*u*CH	7787	7352
No-CH	7198	6813

Age		
Dataset	Hypermethylated in Young	Hypermethylated in Old
3x*u*CH	53	8
No-CH	3	1

Overlap between the two filtering approaches is high for Strain significant CpGs with 13,834 CpGs in common (Euler diagram, Additional file 9: Figure S3). For Age, all four significant ‘No-CH’ CpG sites were present in the ‘3x*u*CH’ significant CpGs. Despite the strong differentiation between strains, there are very few genes that contain CpGs exclusive to either C32 or KA53, and in most cases this is only 1–5 CpG sites. An exception to this is gene LOC116934226, encoding an ATP-dependent RNA helicase me31b-like protein, which had 18 sites hypermethylated in strain KA53 versus C32; 12 in exon three, four in exon four and two in exon five: Of these significant CpGs, only one from exon, four and two from exon, and five occurred within protein coding sequence (Additional file 10: Table S5) with the rest present in the 5′ UTR.

BS-SNPer predicted 317,986 and 424,261 single-nucleotide polymorphisms (SNPs) from the WGBS data in strains C32 and KA53, respectively; of these 30,817 SNPs were exclusive to C32 and 137,092 to KA53. Compared against the reference assembly of South Korean origin, 287,169 SNPs were shared by both United Kingdom collected strains, a further 100,085 of which were fixed in both. Numbers of predicted SNPs were highly similar for the ‘No-CH’ BS-SNPer results (Additional file 7: Table S3).

There were few enriched GO terms relative to the number of genes containing exons with hypermethylated CpGs (Table 2). The category with most GO terms enriched was ‘One or more hypermethylated CpGs’ for biological process (BP) for C32 and KA53 at 21 terms for both (enriched GO terms for the eight comparisons made: Additional file 11: Table S6).

**Table 2:** Number of genes overlapped by significant CpG sites in exonic, coding sequence and intronic regions for all sites and genes with five or more hypermethylated CpGs

Strain	Exonic	CDS	Intronic
C32	2625	1701	1495
KA53	2469	1603	1364
5 or more hypermethylated CpGs			
C32	276	182	94
KA53	292	185	100

### CpGs associated with age and a potential epigenetic clock

Genes overlapping CpG sites significant for Age are given in Additional file 12: Table S7. For the 53 CpGs hypermethylated in Young *Daphnia* in the 3x*u*CH dataset, 35 overlap with gene bodies. All eight CpGs hypermethylated in Old replicates overlap a gene, five of which occur in the coding sequence of a single gene—histone H3 (LOC116920166). For the CpGs hypermethylated in Young individuals, one uncharacterised gene (LOC116933951) is overlapped by three significant CpGs, and nine further genes are overlapped by two significant CpGs, including prefoldin RPB5 interactor-like, homeobox protein Hox-B1a-like, innexin shaking-B-like, and an uncharacterised locus.

**Fig. 3:**
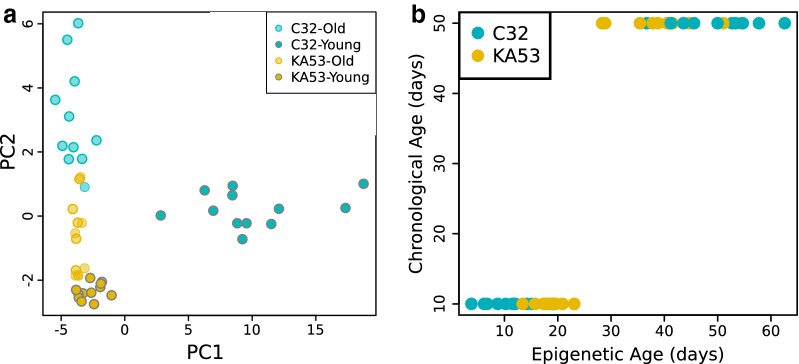
Epigenetic age correlates with chronological age. **a** PCA analysis of beta values for 60 Age significant CpGs (one position was removed as it contained N/A values for certain replicates due to no read coverage) showing that C32 Young individuals cluster separately on PC1. **b** The relationship between predicted epigenetic age and chronological age for all replicates for the ‘3x*u*CH’ dataset, coloured by strain and calculated from the 12 CpGs that contributed most to the penalised lasso regression model

After filtering for CpGs with zero read coverage in any replicate, only one position was removed from the ‘3x*u*CH’ Age significant CpGs (leaving 60 in total), which were analysed by PCA of beta values (the methylated proportion of cytosines at a single CpG, Fig. 3a). Young individuals of strain C32 separated clearly along PC1 which explains 66% of the variance in the data, while PC2 explained 8% of the variance. These 60 positions were input to the penalised lasso regression model, which revealed 12 sites that contributed significantly to the model (Additional file 13: Table S8). The relationship between epigenetic age and chronological age has a Spearman’s rho of 0.87 (*p*-value 3.9 × 10^–15^) per replicate as predicted by these 12 CpGs as shown in Fig. 3b. Strain KA53 had average epigenetic ages of 18.8 and 40.6 days at chronological ages of 10 and 50 days, respectively, whereas for C32 epigenetic ages increased from 11.6 to 49.0 days over the experiment. A GLM of the predicted ages per replicate extracted from the penalised regression indicated that Age explained 81% of the variance in the model, with clone explaining only 0.02% of the variance. However, the Strain by Age interaction explained a moderate amount of variation, 5.7%. (results: Additional file 14: Table S9) in line with Fig. 3a in which Young replicates of C32 appeared distinct from the rest of the experiment. This was also true when only the twelve penalised regression selected CpGs are selected (Additional file 15: Figure S4, part A). The distinction between strains was greater when considering the four Age significant CpGs for the ‘No-CH’ filtered dataset, all of which contributed to the penalised regression (Additional file 13: Table S8). For that regression Strain explained 17% of the variance and was highly significant, as was Age at 34%, and their interaction at 23% (Additional file 13: Table S8 and Additional file 14: Table S9). The effect of strain can be seen in the correlation between chronological and epigenetic age, in which C32 replicates were all younger epigenetically than KA53 replicates at day 10 chronological age (Additional file 15: Figure S4, part B).

## Discussion

### Methylated CpGs were enriched in exons

Methylated CpGs were concentrated in gene bodies with a preference for exons 2 to 5 (Fig. 2d), adding to the evidence that methylation is a directed process in *Daphnia* [8, 9]. We report an average CpG methylation of 1.2–3%, which is higher than our previous estimate for strain C32 of 0.7% [59]. This earlier estimate is more in line with those reported for other strains of *D. magna* at 0.25–0.85% [8, 9, 55–57]. The current higher estimate may result from incomplete bisulphite conversion during library preparation, as the percentage was elevated when filtering out all methylated CHG/CHH sites (the ‘No-CH’ dataset) which occur negligibly in *D. magna* [56]. The CpG methylation rate of the mitochondrial genome has been used as a method of estimating non-conversion rate for *D. magna* before [58], as no mitochondrial DNA methylation occurs in the crustacean *C. gigas* [13]. Our data had a mitochondrial CpG average methylation rate of 0.8% for the ‘3x*u*CH’ and 0.65% for the ‘No-CH’ datasets, which when subtracted from the global CpG percentages (‘3x*u*CH’ dataset: 1.3–0.8% = 0.5%) were in the range of other estimates derived from *D. magna*. This lack of mitochondrial DNA methylation has not been confirmed in *D. magna* or any invertebrates beyond *C. gigas* [13], so the mitochondria method should be interpreted with caution (see [66] for mitochondrial CpG distributions in animals). We argue that by performing a well-replicated experiment in which there are 24 versus 23 replicates for each level of factor tested (Age/Strain/Food), the effect of false-positive CpGs resulting from non-conversion of cytosines was minimised.

### CpG methylation did not respond to caloric restriction

No CpGs were significant for CR at any filtering threshold despite this treatment having an effect on lifespan for KA53 (Fig. 1b) and other phenotypic effects on strain C32 [1–3, 59, 67]. This contrasts with our previous results for a CR-only CpG methylation experiment using C32 [59]. There was no association between median lifespan and CR for the survival analysis in this strain, but it does show previously established maternal effects in response to CR [67]. We believe this experiment to be a more robust test of CR than the previous research for two reasons: (1) our experiment includes more replicates of each food level (24 and 23 in total) and includes two strains; (2) the method used here to identify differentially methylated CpGs makes no assumptions about the underlying distribution of CpGs genome wide. Hearn et al. applied smoothing across linked CpGs to identify differentially methylated regions, an approach which was developed with much higher levels of methylation as a basis [68]. As a result, we recommend DSS-like methods that test significance of an individual CpG for arthropod studies until smoothing approaches that better model underlying CpG distributions in arthropods become available.

The lack of significant CpGs for food is at variance with previous miRNA and protein-coding gene expression experiments exploring responses to CR [1, 3]. Greater than 6000 genes significantly differentially expressed in response to CR in C32 [1], but we found no overlap between gene expression response to CR and the previously identified differentially methylated regions in Ref. [1]. Given this very strong gene expression response, if changes in CpG methylation also formed part of the downstream response to CR in *D. magna,* we might expect to see differential methylation [69]. We conclude that the downstream responses to CR in *D. magna* do not cause changes in DNA methylation.

### Strain-dependent methylation differences were widespread

We showed that two strains of *D. magna* that have > 15,000 differently methylated CpGs for the ‘3x*u*CH’ dataset. This is perhaps not surprising if genetic variability correlates with epigenomic differences as for vertebrates [14, 16]. The maintenance of high-genetic diversity within natural *Daphnia* populations due to clonal lineages that result from extended periods asexual reproduction [42–44] could therefore amplify polymorphism at the sequence *and* methylation level versus sexually recombining species. We see support for this in the 30,817 and 137,092 SNPs exclusive to strains C32 and KA53, respectively. Both strains are distant from the South Korea-derived reference sequence as shown by the 287,169 SNPs shared between strains. These SNP numbers must be considered with care, however, as variant predictions derived from WGBS data are likely to be inflated by false positives from methylation polymorphisms [70, 71]. Strain-specific CpGs are concentrated in gene bodies across thousands of genes (Table 2), and the functional effects of such widespread differential methylation resulting from genetic variation are unknown.

One gene, LOC116934226, hypermethylated strongly at multiple CpGs in strain KA53 exclusively encode ATP-dependent RNA helicase me31B-like protein. *Me31B* is an RNA-binding protein that represses thousands of maternal mRNAs during *Drosophila* maternal-to-zygotic (MZT) transition [72, 73]. MZT is the process by which development comes under zygotic genome control over stored maternal genetic material in animals [74]. It is an essential gene for successful oogenesis and embryogenesis in *Drosophila* [74], with other mRNA-related decay functions that include antiviral activity in mosquitoes [75]. A methylation-dependent difference in embryonic expression levels of *me31b* could change developmental trajectories of embryos of each strain with phenotypic effect. The intergenerational stability of methylation at this locus will be a target of future work, by crossing these strains in future experiments will confirm whether CpG methylation in *D. magna* inheritance is linked to polymorphism.

GO term enrichment was less enlightening, possibly because strain can be considered a random effect within our experiment, albeit with only two levels. This means we do not know if the strains differ ecologically in their original environment, which makes it difficult to correlate general GO terms with phenotype in a biologically meaningful manner.

### Age resulted in DNA methylation changes

For both filtering methods, there were significant CpGs for Age, although a ten-fold difference is observed between the ‘3x*u*CH’ (61 CpGs) and ‘No-CH’ (4 CpGs) datasets. PCA of the ‘3x*u*CH’ Age significant CpGs reveals that Young replicates of strain C32 separate on PCA1 (Fig. 3a), suggesting that Strain can contribute to the Age effect. This is despite the lack of significant Strain by Age interaction CpGs from the DSS results, which may reflect a lack of power to detect such interactions under our experimental design and read coverage levels.

Five of the eight significant CpGs hypermethylated in Old individuals occurred in the histone H3 gene towards the 3′ end of coding sequence. This is the first observation of differential methylation along a histone gene in *Daphnia* as far as we are aware. Histone H3 is one of the five highly conserved histone genes that pack DNA into nucleosomes, and are of fundamental importance to organismal function [7]. It has several post-translational associations with heterochromatin (H3K9me3), activated transcription (H3K4me3/H3K36me3/H3K27ac), gene downregulation (H3K27me3) or a context-dependent effect (H3K4me1) [7]. Post-translational modifications of histones have been shown to differ between sexes in *D. pulex* [9]. Histone modifications are associated with behavioural transitions in ants and are important in head development of lepidoterans [7, 76–78]. From these observations, we predict that Increased methylation of histone H3 may increase this gene’s expression with age with potentially organism-wide functional consequences. In support of this, increased DNA methylation has been shown to increase gene expression in *D. magna* and *D. pulex* [8, 9]. However, this positive relationship between methylation and expression has not been shown to occur within an organism due to a treatment (where ‘ageing’ is the treatment). DNA methylation is not the only regulatory mechanism to differ with age in *D. magna*, as micro-RNA expression differs between young and old individuals and the eggs of young and old, respectively, in strain C32 [3]. Any interactions between miRNAs and constituents of chromatin, like histone H3, in arthropods have not yet been elucidated [7, 79] in contrast to the piwi-interacting RNAs [80], class of small RNAs.

Although more sites are significantly hypermethylated in young individuals, no gene or genes emerge with potentially large effects according to their function. Only uncharacterised gene ‘LOC116933951′ has more than two overlapping significant CpGs; it encodes an integrase domain and is present on a short contig of 5139 bp length. This gene is likely a repetitive element that may have multiple genomic copies, which is supported by an average read coverage of 2426 for CpGs for data from all replicates combined (‘3x*u*CH’ dataset) versus 661 for CpGs placed onto chromosomes. The lack of pattern in sites losing methylation with age could result from reduced efficiency of methylation maintenance protein DNMT1 [81], which is associated with lower expression with increasing age in humans [82]. Our sampling point of 50 days for Old replicates was constrained by a requirement for enough individuals to obtain sufficient DNA input for whole-genome bisulphite sequencing (WGBS). *D. magna* individuals can survive for longer [1, 83], and if DNMT becomes less efficient through lowered expression with age, we predict more hypomethylation at the same, and possibly more, CpG sites. Such studies are becoming feasible with newer methods exploring DNA methylation, especially when candidate CpG sites are targeted [26].

### Potential for an epigenetic clock in a model invertebrate

In an exploratory analysis, we applied penalised lasso regression to the Age significant CpGs of the ‘3x*u*CH’ dataset which identified 12 CpGs contributing significantly to the model specified. The interaction between Age and Strain was significant, but modest (6%) compared to that of Age alone (81%) at these CpGs Predicted or ‘epigenetic ages’ based on these twelve sites that were highly correlated with chronological age for replicates of both strains. (Fig. 3b). All 4 sites significant for the ‘No-CH’ dataset were kept by the model, but these positions were more strongly influenced by Strain, as young replicates of each clone had distinct epigenetic ages (Additional file 15: Figure S4, part B). This is despite the initial DSS analysis finding no CpGs with significant interactions between factors, possibly due to low power as millions of positions were included. Our results are the first, tentative, step in exploring clock-like mechanisms in an invertebrate. The twelve and 4 sites predicted here by the ‘3x*u*CH’ and ‘No-CH’ datasets, respectively, require validation in multiple strains of *D. magna* for assessing whether a species-wide epigenetic clock is feasible. We plan to do this in future work, following the approach of Little et al. [26] for wild mice. The availability of such epigenetic age markers in tandem with the finely controlled life-history experiments commonly performed on *D. magna* has great potential for the field.

## Conclusions

In summary, we found (1) no effect of caloric restriction on methylation, in contrast with prior work that used a different analysis approach; (2) more than 15,000 CpGs differentially methylated between strains sampled from the same habitat; and (3) that Age is associated with 61 differentially methylated CpGs, and some of these sites could prove useful for developing an epigenetic clock. Functional inferences are limited for the effect of Strain and Age on phenotype, but for each of these two factors, a single-gene emerges that could have a widespread phenotypic effect. For Strain, Me31b is a key embryonic repressor of maternal mRNAs, differential methylation of which leading to changed expression levels is hypothesised to put each strain on modified developmental trajectories. For Age, the hypermethylation of histone H3 may change expression levels between Young and Old individuals very broadly, as we have seen before for miRNAs. It is also a point of regulation between two epigenetic mechanisms, DNA methylation and chromatin, that has not been reported previously in *Daphnia*. Future work will focus for (2) on incorporating more strains from different locations to test whether epigenetic variation is more diverse within, or between, habitats. For (3) we now have a target set of CpG sites that we will test for clock-like behaviour in other strains of *D. magna*. The potential for an epigenetic clock in an easily manipulated, relatively short-lived, life-history trait model invertebrate for ageing research is very exciting.

## Methods

### *D. magna* ageing and caloric restriction survival analysis

Both strains originate from Kaimes pond near Leitholm, Scottish Borders, United Kingdom [84]. The experimental conditions specified below were the same as for the survival analysis presented in Ref. [1], and the results for strain C32 were first published in that study and reproduced here for comparison with KA53. Prior to the experiments, replicates of each strain were put through three generations of acclimation to harmonize environmental effects. During this period, each individual was maintained in a 60‐ml glass jar filled with artificial pond medium [85] which was changed twice a week and after the birth of a clutch. Each individual was fed ~ 6.25 × 10^6^ *Chlorella vulgaris* cells daily and was maintained on a 12:12 h Light:Dark cycle at 20 °C. Offspring from the second clutch initiated each generation and the experimental generation. We define Caloric restriction as approximately 20% of normal food levels. From acclimated females of each strain, two offspring were taken, and one replicate was given normal food of ~ 6.25 × 10^6^ cells and the other given a calorically restricted ~ 1.4 × 10^6^ cells. Each food treatment and genotype combination were replicated 24 times and date of birth and date of death of all individuals in the experiment were recorded.

CR-induced differences in lifespan difference within each strain were tested by a Cox's proportional hazards model using the survival package (R code, Additional file 16, strain longevity data: Additional file 17) [86]. Between strain differences were not tested as the experiments were run separately, but under identical conditions. The response variable was days alive and the explanatory variable was food level. We extracted the median day of survival at each food level, and associated p-value from a likelihood ratio test of food levels.

### *D. magna* Ageing and CR experiments for WGBS

Maternal lines of each strain were acclimatised identically to the survival analysis above. Following three generations of acclimatisation, 20 offspring from each mother were isolated to form a biological replicate. We generated six replicates per age and food level for both clones, resulting in 24 replicates per clone and 48 replicates in the experiment in total (summarised in Fig. 1 b). Twelve replicates per clone were fed a normal diet of 5 × 10^6^ algal cells/day and twelve were fed a CR diet of 1 × 10^6^ algal cells/day (as for [3, 59], but differing slightly from the survival analysis). Each replicate was split and reared in four sub-replicate jars of five animals, which were subsequently pooled for DNA extraction. Six replicates of the twelve at each food level and clone were harvested after the first clutch at approximately 10 days (forming ‘Young’ replicates) and the other six after the fifth clutch at approximately 50 days (‘Old’ replicates). *D. magna* were ground by motorised pestle in Digsol and proteinase K and incubated overnight at 37 °C and stored at − 70 °C until DNA extraction. DNA was extracted from pooled *D. magna* per replicate by phenol–chloroform followed by a Riboshredder RNA digestion step and repeat of the phenol–chloroform extraction (following [59]). DNA was eluted into 100 μl of TE buffer and quantified by Qubit fluorimeter by dsDNA HS Assay Kit. Sample purity was checked by Nanodrop 260:280 and 260:230 and DNA integrity was examined by running approximately 35 ng DNA on a 0.8% agarose gel stained with ethidium bromide.

### Methylated CpG and variant prediction

Bisulphite libraries for WGBS were prepared using EpiGnome/TruSeq DNA methylation kits and 150 bp paired-end sequenced on Illumina HiSeq 4000 by the Centre for Genomic Research (Liverpool, United Kingdom), and raw data deposited in the European Nucleotide Archive (PRJEB34509). Reads were provided by CGR post-trimming with Cutadapt v1.2.1 and Sickle v1.200 for adapters and read quality. Cutadapt option-O 3 was used to trim the 3′ end of any reads which matched the adapter sequence for 3 bp, and Sickle removed read windows with an average phred-scaled quality score below 20. Reads shorter than 20 bp after trimming were removed.

Read quality was assessed with FastQC, and the first 15 bp of each read trimmed with fastp v0.19.3 to remove biases introduced by the random-priming library preparation step, and processed reads re-assessed with FastQC. We applied the Bismark (v0.22.3) pipeline (with options “-N 1 –score_min L,0,-0.6”) to generate CpG methylation calls for each replicate using the chromosomal *Daphnia magna* (KIT strain) genome assembly version as a reference [64]. Bismark mapping rates were compared using Spearman’s Rho (in base R 4.0.0) between this reference and *D. magna* assembly 2.4, which was the reference used in our previous work [59]. Aligned reads were then deduplicated in Bismark, and we evaluated three filtering methods to reduce the effect of bisulphite reaction non-conversion on methylation calls. Firstly, all reads with methylation at non-CpG sites were removed, this allowed direct comparison with Hearn et al. 2019 and we did this as non-CpG methylation (at CHH and CHG positions) is considered negligible in *D. magna* [55]—referred to as the ‘No-CH’ dataset throughout. Secondly, we filter reads with three or more consecutive unconverted CH cytosines as recommended for BS datasets by [63], which we refer to as the ‘3x*u*CH’ dataset here. Both of these methods were implemented through the ‘*filter_non_conversion*’ tool within Bismark, and the results for unfiltered methylation calls are presented for comparison with the two filtering methods. Finally, CpG read coverages for methylated and unmethylated reads were combined for the forward and reverse strand data per CpG using the Bioconductor package bsseq [68] (R code, Additional file 16). Mapping rates and other metrics per replicate were generated in multiQC [87]. To assess inter-strain genetic differences, SNPs were called in BS-SNPer (v1.0) [70] on ‘*filter_non_conversion*’ output bam files pooled for each strain (minus sample 5 for C32) with parameters: ‘–minhetfreq 0.1 –minhomfreq 0.85 –minquali 15 –mincover 10 –maxcover 1000 –minread2 2 –errorate 0.02 –mapvalue 20’. SNP calls were filtered at phred-scaled quality 20 and intersected in bcftools (*isec*, v1.11).

Clustering of replicates was explored by principal components analysis (PCA) in CPGTools [88], and hierarchical clustering using the R package pvclust [89] of the 10,000 most variable CpG sites (R code, Additional file 16) with 10,000 bootstrap re-samplings. To do this, methylation status at each position for the ‘3x*u*CH’ dataset was converted into a proportion or *beta* value and sites with zero methylated cytosines or with no read coverage in a replicate were removed; variances were then ranked per methylated position with *beta_topN.py* of CPGTools. To explore genome-wide patters of methylation CpG counts were combined using the ‘3x*u*CH’ dataset and summarised in *CpG_anno_position* of CPGTools for CpG sites with greater than 5% methylation (input gene coordinate BED file: Additional file 18).

### Differential methylation testing

To test the effect of ageing, nutrition, strain and any interactions between them on CpG methylation sites, a generalised linear modelling approach through the Bioconductor package DSS (version 2.36.0 in R version 4.0.0) was developed. Bismark output coverage per replicate was first converted to DSS input format and filtered so that at least two of the six replicates per category (Age × Food × Strain) had a coverage of at least two prior to model testing of all factors and their interactions (~ Strain + Food + Age + Strain:Food + Strain:Age + Age:Food + Strain:Food:Age; DSS R script, Additional file 16). CpG sites with an adjusted p-value of less than 0.05 were considered significant. Gene ontology (GO) term enrichment was tested for genes with exons (coding sequence plus untranslated regions) overlapping hypermethylated CpG sites in each strain. Gene ontology annotations were assigned by eggNOG (version 2.0.1) with the predicted protein sequences of the KIT assembly [64] as input. Enrichment was performed in topGO (version 2.40.0) for biological process (BP) and molecular function (MF) categories (R code, Additional file 16) for genes with at least one hypermethylated CpG within exons and the subset of genes with least five hypermethylated CpGs per exonic sequences. The ‘weight01′ algorithm and a p-value threshold of less than 0.01 were applied; p-values were not corrected in line with topGO user manual recommendation (see topGO user manual Sect. 6.2: The adjustment of p-values).

### Penalised regression of age significant CpG sites

To explore the potential for an epigenetic clock in *D. magna,* we performed a penalised logistic regression on the Age significant CpGs on per replicate beta values implemented using the caret and glmnet R packages [90, 91] (R code, Additional file 16). A lasso (or Least Absolute Shrinkage and Selection Operator) regression was applied to identify coefficients, in this case Age significant CpGs, that contribute most significantly to the model in which Age is the explanatory variable, leaving us with the best CpG candidates for constructing a clock. The predicted or ‘epigenetic age’ of each replicate according to these CpGs was extracted from the model and plotted against chronological age (10 or 50 days). Finally, we tested if these epigenetic ages were predicted by Age, Strain and their interaction (Predicted Age ~ Strain + Age + Strain:Age) through a generalised linear model (GLM code, Additional file 16). We extracted effect sizes for Age and Strain by dividing the sum of squares for each factor and their interaction by the total sum of squares. Effect sizes were multiplied by 100 to give percentage of variance explained.

## Supplementary Information

**Additional file 1.** MultiQC results for the ‘3xuCH’ filteressd datasets in html format.

**Additional file 2.** MultiQC results for the ‘No-CH’ filtered datasets in html format.

**Additional file 3: Figure S1.** PCA plot of replicates for principal components one and two for the top 10000 most variably methylated CpGs by beta value including sample 5. This sample originates from C32 but clusters with KA53, it represents a mislabelled or contaminated sample and was removed from further analysis; C32 = red, KA53 = green.

**Additional file 4: Table S1.** Metrics for 5% plus CpG methylated sites across gene-bodies, generated in CpGTools and used to create Fig. 2c).

**Additional file 5: Figure S2.** Distribution of CpGs with greater than 5% methylation combined across gene-bodies and by exon number (1 = closest to transcription start site) separately for each strain, showing that the distributions do not differ between them.

**Additional file 6: Table S2.** Metrics for 5% plus CpG methylated sites across exons, generated in CpGTools and used to create Fig. 2d).

**Additional file 7: Table S3**. Number of DSS significant CpG sites for each factor and their interactions at each filtering level and number of SNPs predicted in each strain by BS-SNPer.

**Additional file 8: Table S4.** DSS output for significant CpGs for Age and Strain for ‘3xuCH’ and ‘No-CH’ datasets. Each category occurs consecutively in the file.

**Additional file 9: Figure S3.** Euler diagram of the overlap between ‘3x*u*CH’ and ‘No-CH’ filtering for CpGs significantly differentially methylated between strains.

**Additional file 10: Table S5.** Hypermethylated positions in strain KA53 for gene LOC116934226 by exon.

**Additional file 11: Table S6.** Enriched GO terms for one or five plus enriched CpGs hypermethylated in each strain for Biological Process and Molecular Function GO categories (3x*u*CH dataset).

**Additional file 12: Table S7.** Gene annotations for genes overlapping CpGs significantly associated with ageing in *D. magna*.

**Additional file 13: Table S8.** CpGs with a significant contribution to epigenetic age and their coefficients after penalised lasso regression for ‘3x*u*CH’ and ‘No-CH’ datasets.

**Additional file 14: Table S9.** Summary table of GLM results for predicted epigenetic age against Age and Strain ‘3x*u*CH’ and ‘No-CH’ datasets.

**Additional file 15: Figure S4.** Part A) PCA analysis of beta values for the twelve Age significant CpGs selected by penalised lasso regression. As for Fig. 3a), C32 young individuals cluster separately on PC1. Part B) The relationship between predicted epigenetic age and chronological age for all replicates for the ‘No-CH’ dataset, coloured by strain and calculated from the 4 CpGs that contributed most to the penalised lasso regression model.

**Additional file 16.** R code for combining complementary CpGs, Cox’s proportional hazards, DSS analysis, topGO analysis, and penalised regression.

**Additional file 17.** Longevity experiment results for the two strains under caloric restriction and normal food input to the Cox’s proportional hazards analyses, which were run separately for each strain. H = normal food individual; L = caloric restricted individual. Individuals were changed to status ‘2′ on death in line with survival R program requirements.

**Additional file 18.** Gene component coordinates bed file for the KIT1 *D. magna* genome assembly. Used to calculate CpG rates per kb for each category shown in Fig. 2c, d.

## Data Availability

The sequencing reads generated for this study are available in the European Nucleotide Archive under accession PRJEB34509, https://www.ebi.ac.uk/ena/browser/view/PRJEB34509 [92].

## Abbreviations

3x*u*CH: Dataset for which reads with three or more consecutive unconverted CH cytosines were removed prior to differential methylation testing
bp: Base pairs
BP: Biological process
CHH/CHG: Cytosine followed by nucleotides where H is adenine, thymine or cytosine and G is guanine
CI: Confidence interval
CpG: Cytosine followed by guanine
CR: Caloric restriction
DNA: Deoxyribonucleic acid
DNMT: DNA methyltransferase
DSS: Dispersion shrinkage for sequencing data
ENA: European Nucleotide Archive
FDR: False discovery rate
GLM: Generalised linear model
GO: Gene ontology
lasso: Least Absolute Shrinkage and Selection Operator
MF: Molecular function
MZT: Maternal to Zygotic
NF: Normal food
No-CH: Dataset for which reads with all reads containing CH-methylated cytosines were removed prior to differential methylation testing
PCA: Principal components analysis
PC1/2: Principal component 1 or 2
PCR: Polymerase chain reaction
SNP: Single-nucleotide polymorphism
TE: Tris(hydroxymethyl)aminomethane ethylenediaminetetraacetic acid
UTR: Untranslated region
WGBS: Whole-genome bisulphite sequencing
