# Supplementary material for: DNA methylation differs extensively between strains of the same geographical origin and changes with age in Daphnia magna
Source: Epigenetics Chromatin. 2021 Jan 6;14:4. doi: 10.1186/s13072-020-00379-z (PMC7789248; doi:10.1186/s13072-020-00379-z)
Supplement: Supplementary file 1 — Additional file 1. MultiQC results for the ‘3xuCH’ filteressd datasets in html format. [file 13072_2020_379_MOESM1_ESM.html]

MultiQC Report


# Toggle navigation v1.9

Loading report..

- General Stats
- Bismark
  - Alignment Rates
  - Deduplication
  - Strand Alignment
  - Cytosine Methylation
  - M-Bias

Toolbox

### MultiQC Toolbox

#### Apply Highlight Samples

+

Regex mode off
help
 Clear

#### Apply Rename Samples

+

Click here for bulk input.

Paste two columns of a tab-delimited table here (eg. from Excel).

First column should be the old name, second column the new name.

Add

Regex mode off
help
 Clear

#### Apply Show / Hide Samples

Hide matching samples

Show only matching samples

+

Regex mode off
help
 Clear

#### Export Plots

- Images
- Data

px

px

Aspect ratio

PNG
JPEG
SVG

Plot scaling

X

Download the raw data used to create the plots in this report below:

Format:

Tab-separated
Comma-separated
JSON

Note that additional data was saved in `multiqc_data_2` when this report was generated.

---

##### Choose Plots

 All
 None

---


   Download Plot Images

If you use plots from MultiQC in a publication or presentation, please cite:

> **MultiQC: Summarize analysis results for multiple tools and samples in a single report**  
> *Philip Ewels, Måns Magnusson, Sverker Lundin and Max Käller*  
> Bioinformatics (2016)  
> doi: 10.1093/bioinformatics/btw354  
> PMID: 27312411

#### Save Settings

You can save the toolbox settings for this report to the browser.

 Save


---

#### Load Settings

Choose a saved report profile from the dropdown box below:

[ select ]

Load
 Delete
 Set default
 Clear default

#### About MultiQC

This report was generated using MultiQC, version 1.9

You can see a YouTube video describing how to use MultiQC reports here:
https://youtu.be/qPbIlO\_KWN0

For more information about MultiQC, including other videos and
extensive documentation, please visit http://multiqc.info

You can report bugs, suggest improvements and find the source code for MultiQC on GitHub:
https://github.com/ewels/MultiQC

MultiQC is published in Bioinformatics:

> **MultiQC: Summarize analysis results for multiple tools and samples in a single report**  
> *Philip Ewels, Måns Magnusson, Sverker Lundin and Max Käller*  
> Bioinformatics (2016)  
> doi: 10.1093/bioinformatics/btw354  
> PMID: 27312411

# 

A modular tool to aggregate results from bioinformatics analyses across many samples into a single report.

#### JavaScript Disabled

MultiQC reports use JavaScript for plots and toolbox functions. It looks like
you have JavaScript disabled in your web browser. Please note that many of the report
functions will not work as intended.

Loading report..

Report
generated on 2020-10-10, 16:00
based on data in:
`/data/home/jhearn1/Daphnia/Ageing_methylation/Genome2019/3xC/reports`

---

×
don't show again

**Welcome!** Not sure where to start?  
Watch a tutorial video
  *(6:06)*

## General Statistics

 Copy table

 Configure Columns

 Sort by highlight

 Plot
Showing 48/48 rows and 5/7 columns.

| Sample Name | % mCpG | M C's | C Coverage | % Dups | M Unique | M Aligned | % Aligned |
| --- | --- | --- | --- | --- | --- | --- | --- |
| 10\_1 | 1.4% | 290.8 | 15.89X | 29.3% | 10.2 | 14.4 | 40.8% |
| 11\_1 | 1.4% | 204.3 | 11.20X | 23.6% | 7.1 | 9.3 | 45.2% |
| 12\_1 | 1.4% | 187.3 | 10.32X | 24.0% | 6.6 | 8.6 | 40.2% |
| 13\_1 | 1.4% | 444.5 | 23.53X | 25.2% | 14.4 | 19.3 | 55.5% |
| 14\_1 | 1.4% | 440.7 | 23.63X | 30.2% | 14.6 | 20.9 | 56.0% |
| 15\_1 | 1.3% | 345.3 | 18.34X | 22.3% | 11.1 | 14.3 | 56.0% |
| 16\_1 | 1.3% | 337.2 | 17.99X | 27.7% | 10.9 | 15.1 | 53.3% |
| 17\_1 | 1.4% | 346.7 | 18.67X | 26.2% | 11.4 | 15.5 | 45.6% |
| 18\_1 | 1.3% | 397.6 | 21.52X | 32.6% | 13.3 | 19.8 | 64.6% |
| 19\_1 | 1.4% | 167.1 | 9.16X | 26.0% | 5.8 | 7.8 | 37.5% |
| 1\_1 | 1.3% | 269.2 | 14.21X | 27.4% | 8.6 | 11.9 | 44.0% |
| 20\_1 | 1.4% | 222.2 | 11.99X | 28.9% | 7.5 | 10.5 | 48.7% |
| 21\_1 | 1.4% | 208.5 | 11.18X | 28.6% | 6.9 | 9.7 | 49.8% |
| 22\_1 | 1.4% | 228.5 | 12.38X | 31.7% | 7.7 | 11.3 | 41.1% |
| 23\_1 | 1.4% | 289.0 | 15.40X | 28.1% | 9.6 | 13.3 | 43.9% |
| 24\_1 | 1.3% | 360.8 | 19.02X | 25.1% | 11.7 | 15.6 | 43.7% |
| 25\_1 | 1.2% | 358.7 | 18.65X | 22.4% | 11.3 | 14.6 | 50.5% |
| 26\_1 | 1.3% | 299.9 | 15.87X | 27.4% | 9.8 | 13.5 | 48.9% |
| 27\_1 | 1.3% | 293.0 | 15.59X | 23.3% | 9.6 | 12.5 | 54.9% |
| 28\_1 | 1.3% | 302.1 | 16.21X | 25.5% | 10.1 | 13.5 | 53.1% |
| 29\_1 | 1.3% | 292.8 | 15.77X | 25.7% | 9.8 | 13.2 | 52.0% |
| 2r-2\_1 | 1.3% | 384.9 | 20.84X | 30.8% | 13.1 | 19.0 | 40.9% |
| 30\_1 | 1.3% | 439.6 | 23.55X | 26.2% | 14.3 | 19.5 | 52.7% |
| 31\_1 | 1.3% | 172.2 | 9.35X | 19.5% | 5.9 | 7.3 | 32.4% |
| 32\_1 | 1.3% | 234.0 | 12.55X | 21.0% | 7.8 | 9.8 | 44.1% |
| 33\_1 | 1.2% | 295.1 | 15.48X | 20.6% | 9.3 | 11.7 | 39.3% |
| 34\_1 | 1.2% | 439.0 | 23.07X | 25.0% | 13.9 | 18.5 | 43.5% |
| 35\_1 | 1.3% | 502.1 | 26.36X | 22.7% | 16.0 | 20.7 | 53.7% |
| 36\_1 | 1.3% | 384.2 | 20.27X | 20.3% | 12.4 | 15.6 | 46.1% |
| 37\_1 | 1.3% | 399.5 | 21.05X | 19.8% | 12.8 | 16.0 | 56.1% |
| 38\_1 | 1.3% | 371.1 | 19.69X | 24.0% | 12.0 | 15.8 | 52.3% |
| 39\_1 | 1.4% | 290.6 | 16.36X | 24.0% | 10.2 | 13.4 | 66.0% |
| 3\_1 | 1.4% | 253.4 | 13.81X | 27.4% | 8.7 | 12.1 | 46.2% |
| 40\_1 | 1.3% | 341.6 | 18.34X | 29.1% | 11.4 | 16.1 | 46.2% |
| 41\_1 | 1.3% | 533.1 | 28.43X | 29.8% | 17.4 | 24.8 | 64.4% |
| 42\_1 | 1.3% | 441.1 | 23.97X | 31.9% | 14.9 | 21.9 | 65.9% |
| 43\_1 | 1.3% | 330.9 | 18.03X | 28.6% | 11.3 | 15.9 | 50.9% |
| 44\_1 | 1.3% | 346.6 | 18.87X | 32.6% | 11.8 | 17.6 | 59.6% |
| 45\_1 | 1.3% | 364.0 | 19.52X | 29.3% | 11.7 | 16.6 | 62.3% |
| 46\_1 | 1.3% | 438.3 | 23.35X | 36.6% | 14.1 | 22.2 | 62.7% |
| 47\_1 | 1.3% | 385.1 | 20.54X | 32.9% | 12.3 | 18.4 | 57.1% |
| 48\_1 | 1.3% | 382.3 | 20.38X | 32.7% | 12.2 | 18.2 | 56.5% |
| 4\_1 | 1.4% | 206.5 | 11.22X | 27.1% | 7.1 | 9.7 | 44.1% |
| 5\_1 | 1.3% | 241.7 | 13.12X | 28.4% | 8.3 | 11.7 | 52.8% |
| 6\_1 | 1.4% | 200.4 | 10.81X | 29.3% | 6.8 | 9.7 | 31.4% |
| 7\_1 | 1.3% | 159.8 | 8.61X | 25.2% | 5.5 | 7.4 | 34.4% |
| 8\_1 | 1.3% | 195.0 | 10.55X | 25.7% | 6.6 | 9.0 | 35.8% |
| 9\_1 | 1.4% | 156.9 | 8.74X | 23.6% | 5.6 | 7.4 | 29.8% |

×

#### General Statistics: Columns

Uncheck the tick box to hide columns. Click and drag the handle on the left to change order.

Show All
Show None

| Sort | Visible | Group | Column | Description | ID | Scale |
| --- | --- | --- | --- | --- | --- | --- |
| || |  | Bismark | % mCpG | % Cytosines methylated in CpG context | `percent_cpg_meth` | None |
| || |  | Bismark | M C's | Total number of C's analysed, in millions | `total_c` | None |
| || |  | Bismark | C Coverage | Cyotosine Coverage | `C_coverage` | None |
| || |  | Bismark | % Dups | Percent Duplicated Alignments | `dup_reads_percent` | None |
| || |  | Bismark | M Unique | Deduplicated Alignments (millions) | `dedup_reads` | read\_count |
| || |  | Bismark | M Aligned | Total Aligned Sequences (millions) | `aligned_reads` | read\_count |
| || |  | Bismark | % Aligned | Percent Aligned Sequences | `percent_aligned` | None |

Close

## Bismark

Bismark is a tool to map bisulfite converted sequence reads and determine cytosine methylation states.

### Alignment Rates

Number of Reads
Percentages

loading..

---

### Deduplication

Number of Reads
Percentages

loading..

---

### Strand Alignment

All samples were run with `--directional` mode; alignments to complementary strands (CTOT, CTOB) were ignored.

Number of Reads
Percentages

loading..

---

### Cytosine Methylation

loading..

---

### M-Bias

This plot shows the average percentage methylation and coverage across reads. See the
bismark user guide
for more information on how these numbers are generated.

CpG R1
CHG R1
CHH R1
CpG R2
CHG R2
CHH R2

loading..

**MultiQC v1.9**
- Written by Phil Ewels,
available on GitHub.

This report uses HighCharts,
jQuery,
jQuery UI,
Bootstrap,
FileSaver.js and
clipboard.js.

×

### Plot Table Data

Select Column

Select Column

Please select two table columns.

Close

×

### Regex Help

Toolbox search strings can behave as regular expressions (regexes). Click a button below to see an example of it in action. Try modifying them yourself in the text box.

`^` (start of string)
`$` (end of string)
`[]` (character choice)
`\d` (shorthand for `[0-9]`)
`\w` (shorthand for `[0-9a-zA-Z_]`)
`.` (any character)
`\.` (literal full stop)
`()` `|` (group / separator)
`*` (prev char 0 or more)
`+` (prev char 1 or more)
`?` (prev char 0 or 1)
`{}` (char num times)
`{,}` (count range)

```
samp_1
samp_1_edited
samp_2
samp_2_edited
samp_3
samp_3_edited
prepended_samp_1
tmp_samp_1_edited
tmpp_samp_1_edited
tmppp_samp_1_edited
#samp_1_edited.tmp
samp_11
samp_11111
```

See regex101.com for a more heavy duty testing suite.

Close
