# Supplementary figures and images for: DNA methylation differs extensively between strains of the same geographical origin and changes with age in Daphnia magna
Source: Epigenetics Chromatin. 2021 Jan 6;14:4. doi: 10.1186/s13072-020-00379-z (PMC7789248; doi:10.1186/s13072-020-00379-z)

PCA 2D map

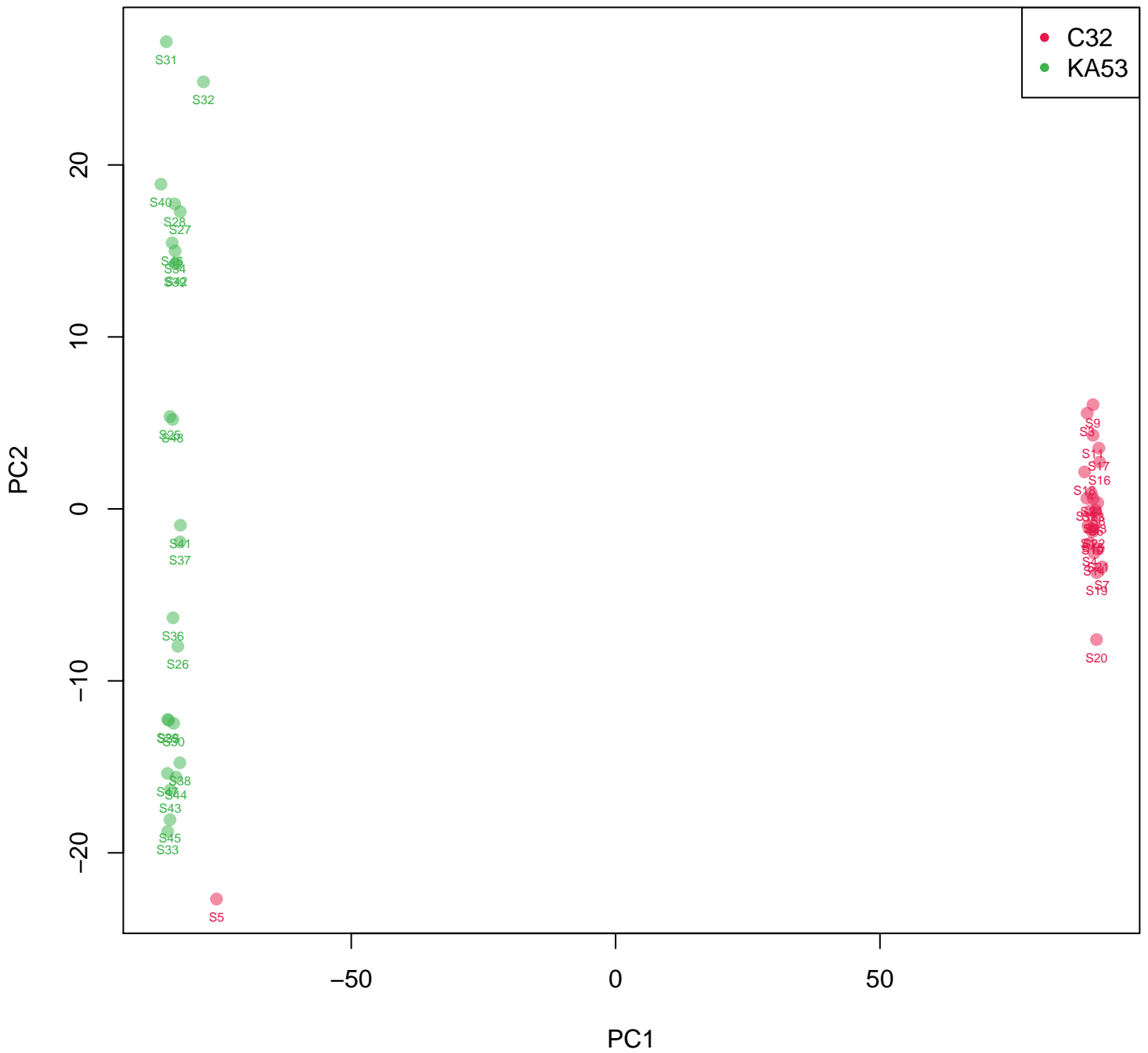

Supplement: Supplementary file 3 — Additional file 3: Figure S1. PCA plot of replicates for principal components one and two for the top 10000 most variably methylated CpGs by beta value including sample 5. This sample originates from C32 but clusters with KA53, it represents a mislabelled or contaminated sample and was removed from further analysis; C32 = red, KA53 = green. [file 13072_2020_379_MOESM3_ESM.pdf]

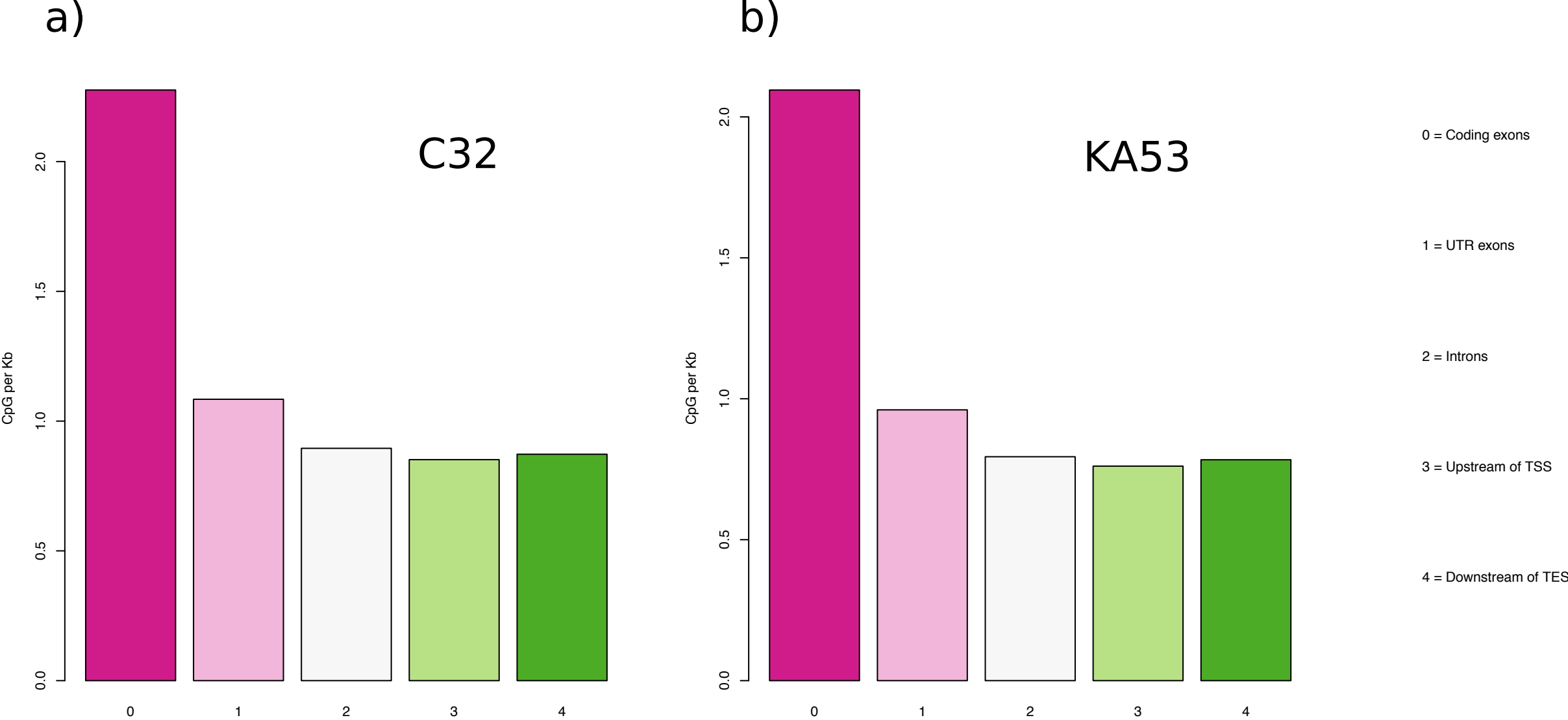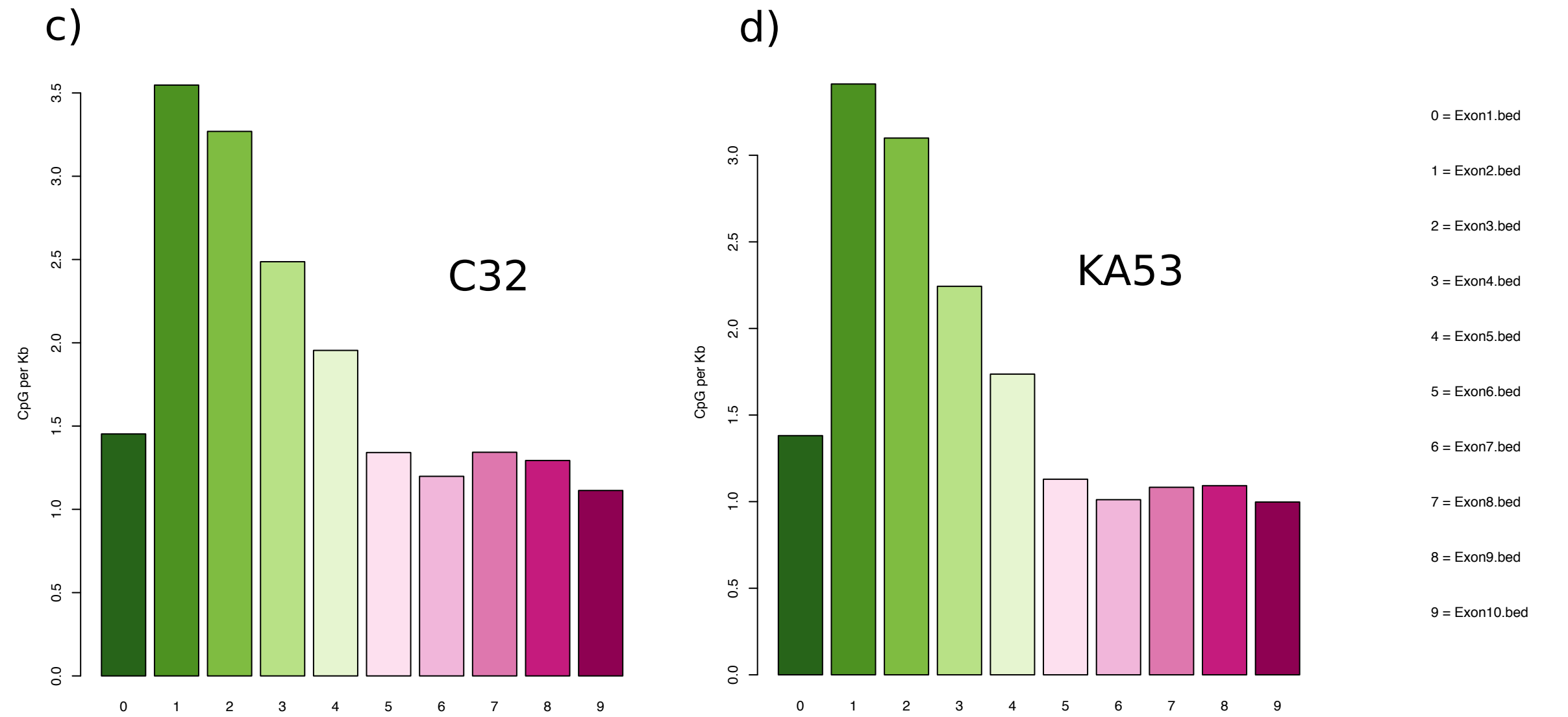

Supplement: Supplementary file 5 — Additional file 5: Figure S2. Distribution of CpGs with greater than 5% methylation combined across gene-bodies and by exon number (1 = closest to transcription start site) separately for each strain, showing that the distributions do not differ between them. [file 13072_2020_379_MOESM5_ESM.pdf]

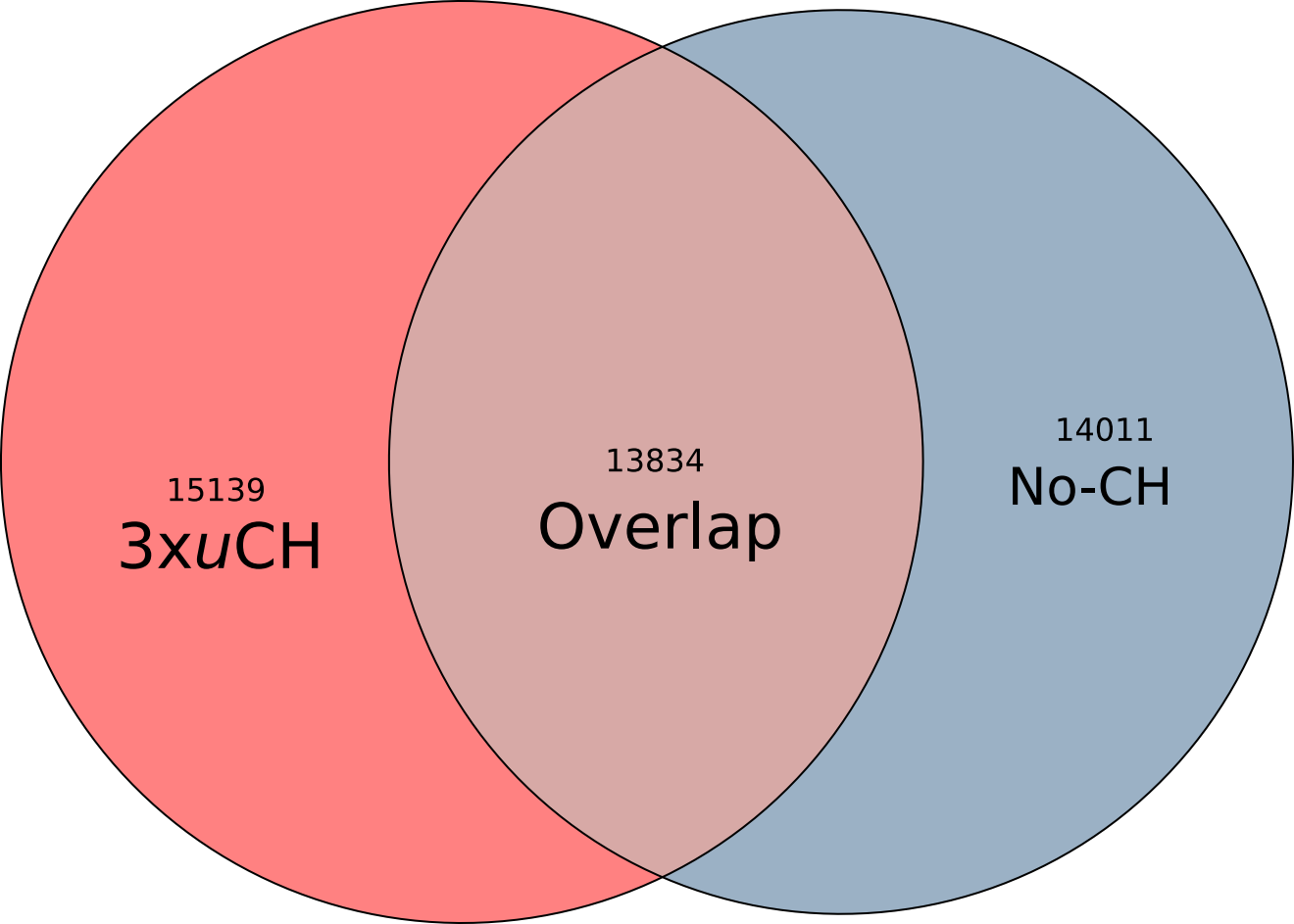

15139  
**3xuCH**

13834  
**Overlap**

14011  
**No-CH**

Supplement: Supplementary file 9 — Additional file 9: Figure S3. Euler diagram of the overlap between ‘3xuCH’ and ‘No-CH’ filtering for CpGs significantly differentially methylated between strains. [file 13072_2020_379_MOESM9_ESM.pdf]

A)

PCA 2D map

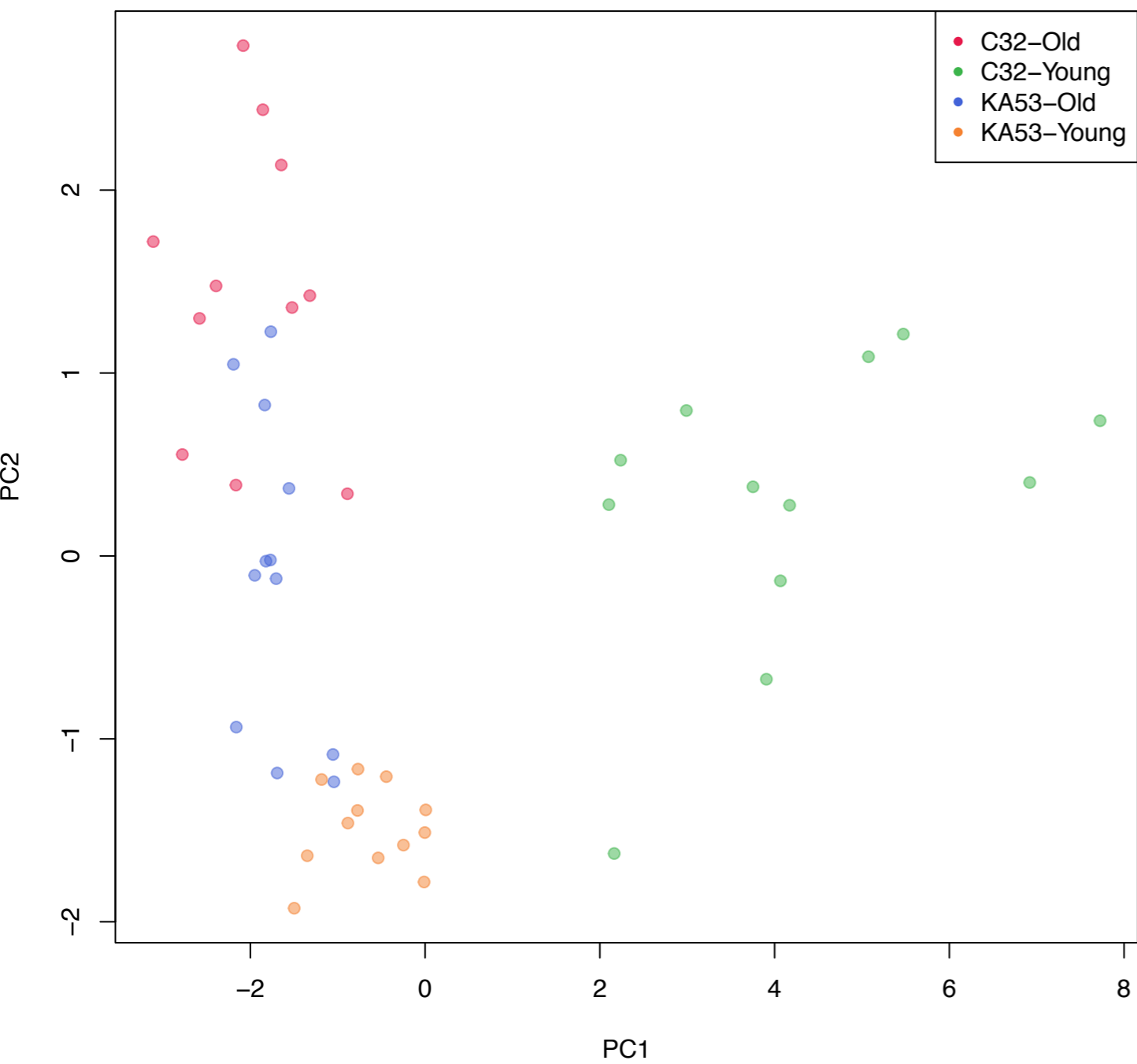

B)

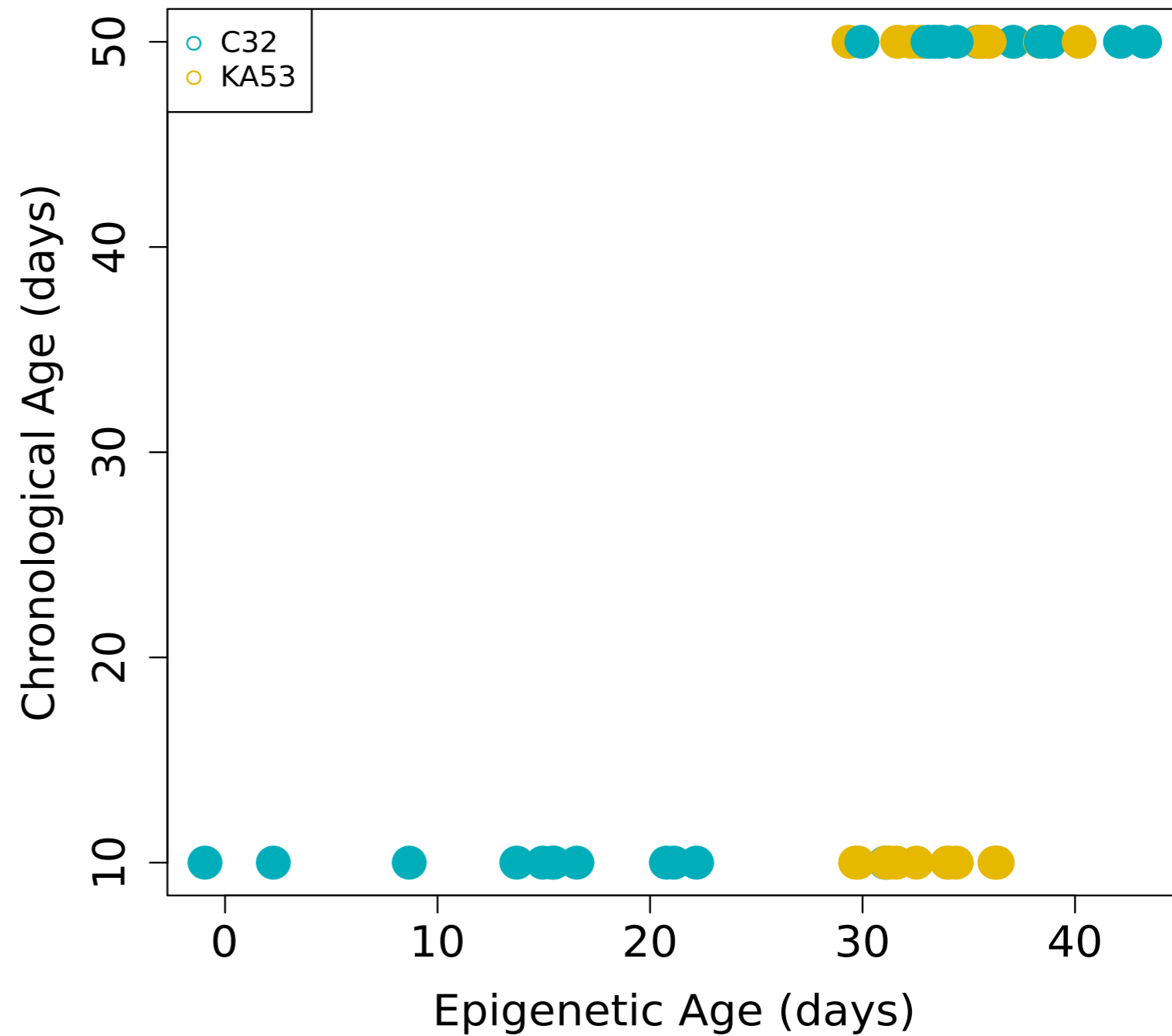

Supplement: Supplementary file 15 — Additional file 15: Figure S4. Part A) PCA analysis of beta values for the twelve Age significant CpGs selected by penalised lasso regression. As for Fig. 3a), C32 young individuals cluster separately on PC1. Part B) The relationship between predicted epigenetic age and chronological age for all replicates for the ‘No-CH’ dataset, coloured by strain and calculated from the 4 CpGs that contributed most to the penalised lasso regression model. [file 13072_2020_379_MOESM15_ESM.pdf]
